# Functional ultrasound imaging of intrinsic connectivity in the living rat brain with high spatiotemporal resolution

**DOI:** 10.1038/ncomms6023

**Published:** 2014-10-03

**Authors:** Bruno-Félix Osmanski, Sophie Pezet, Ana Ricobaraza, Zsolt Lenkei, Mickael Tanter

**Affiliations:** 1 Institut Langevin, ESPCI-ParisTech, 1 rue Cuvier, 75005 Paris, France; 2 CNRS UMR 7587, 1 rue Cuvier, 75005 Paris, France; 3 INSERM U979 ‘Wave Physics for Medicine’ Lab, 1 rue Cuvier, 75005 Paris, France; 4 Centre National pour la Recherche Scientifique, UMR 8249, 10 rue Vauquelin, 75005 Paris, France; 5 Brain Plasticity Unit, ESPCI-ParisTech, 10 rue Vauquelin, 75005 Paris, France

## Abstract

Long-range coherences in spontaneous brain activity reflect functional connectivity. Here we propose a novel, highly resolved connectivity mapping approach, using ultrafast functional ultrasound (fUS), which enables imaging of cerebral microvascular haemodynamics deep in the anaesthetized rodent brain, through a large thinned-skull cranial window, with pixel dimensions of 100 μm × 100 μm in-plane. The millisecond-range temporal resolution allows unambiguous cancellation of low-frequency cardio-respiratory noise. Both seed-based and singular value decomposition analysis of spatial coherences in the low-frequency (<0.1 Hz) spontaneous fUS signal fluctuations reproducibly report, at different coronal planes, overlapping high-contrast, intrinsic functional connectivity patterns. These patterns are similar to major functional networks described in humans by resting-state fMRI, such as the lateral task-dependent network putatively anticorrelated with the midline default-mode network. These results introduce fUS as a powerful novel neuroimaging method, which could be extended to portable systems for three-dimensional functional connectivity imaging in awake and freely moving rodents.

## Abstract (web summary)

Functional connectivity of brain networks is poorly understood, in part, due to limited imaging approaches. Here, the authors use ultrasound imaging to study functional connectivity in the adult rat brain *in vivo*, allowing for the identification of highly contrasted intrinsic connectivity patterns.

## Graphical abstract

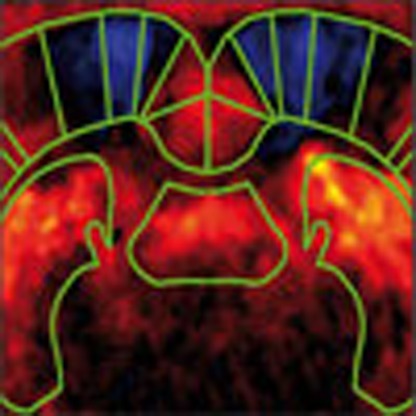

The brain dynamically integrates and coordinates responses to internal and external stimuli across multiple spatiotemporal scales through large-scale functional networks. Assessment of its functional connectivity (FC), through the measurement of regionally correlated, spontaneous, low-frequency (0.01–0.1 Hz) fluctuations in blood oxygen level-dependent (BOLD) signals with functional magnetic resonance imaging (fMRI), particularly during resting-state/task-free periods (resting-state fMRI or rsfMRI)^1^, has greatly advanced our understanding of the functional organization of the human brain^2^. Intrinsic connectivity networks, such as the default-mode network^3^,^4^, ventral and dorsal attention networks^5^,^6^, and salience network^7^, have been intensely studied in both basic and clinical cognitive neuroscience fields. These correlated resting-state BOLD fluctuations appear to be a fundamental property of the brain because they are present during sleep^8^ and even during general anaesthesia^9^. Indeed, these fluctuations are temporally coherent among brain areas that are structurally connected and functionally related^1^,^10^,^11^. Most, if not all, neurological and psychiatric diseases involve the disruption of large-scale functional and/or structural properties in the brain^11^,^12^. These include major pathologies such as schizophrenia, depression and Alzheimer’s disease. Consequently, investigation of the FC under well-controlled and experimentally accessible conditions is of major scientific interest, because it may lead to better diagnostic or prognostic indicators, and more targeted and controlled drug treatments. Therefore, the development of the corresponding translational rodent models is also of great interest. Indeed, recent reports show the presence of prominent intrinsic networks in the monkey^9^ and rat brain^13^.

To date, only rsfMRI has been able to image intrinsic brain networks with the appropriate spatial resolution and coverage. However, the low-frequency oscillations measured in rsfMRI studies can be contaminated by higher frequency (>1 Hz) physiological noise, such as the cardiac cycle and respiratory motion^14^,^15^. Therefore, the unambiguous and general exclusion of physiological noise requires novel techniques that are able to temporally resolve signals above 1 Hz. Magnetic resonance imaging (MRI) has additional technical and logistic challenges, such as the necessity for high magnetic fields, problems with motion artefacts, electromagnetic compatibility issues, high costs and lack of portability. These concerns currently hinder the broad dissemination of FC research in translational and pre-clinical research settings. Therefore, the validation of complementary or alternative methods for *in vivo* imaging of intrinsic FC is an important scientific objective. Recently, very high frame rate ultrasound imaging (>10,000 frames per second) was shown to enable high-resolution and high-sensitivity power Doppler imaging of cerebral blood flow (CBF)^16^, leading to functional ultrasound (fUS) imaging of task-evoked changes in cortical activity^17^. However, this study used highly invasive craniotomy, which is not optimal for the imaging of resting-state functional networks. Indeed, craniotomy was shown to induce important behavioural, morphological, biochemical and vascular changes in the rat brain^18^,^19^,^20^. In consequence, currently we do not know whether fUS is capable of mapping intrinsic FC patterns and we do not know the relative performance of this method as compared with the standard fMRI-based approach.

Here we develop a large thinned-skull cranial window preparation and an ultrasonic sequence for ultrafast imaging of intrinsic FC of the living adult rat brain. This approach enables us to identify highly contrasted intrinsic FC patterns. Our approach represents a simple, portable, cost- and space-effective, highly resolved, and motion and pulsatility artefact-free method, capable of imaging FC deep in the rodent brain.

## Results

### Identification of putatively interconnected seed areas

**Figure 1:**
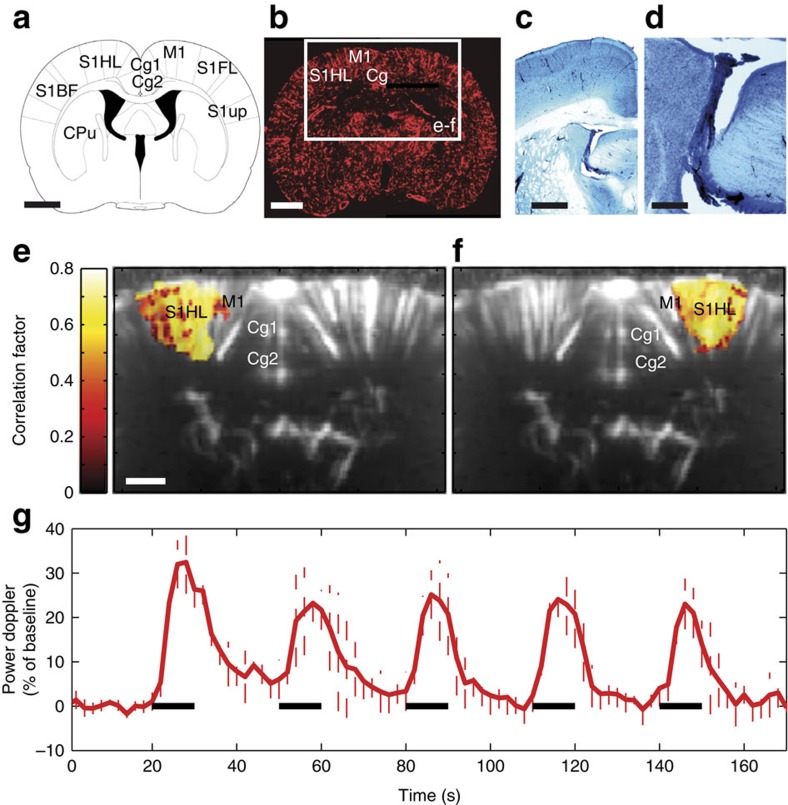
Functional identification of putatively interconnected seed regions using task-evoked haemodynamic changes. Anatomical organization of the rat brain at Bregma −0.6 mm (**a**) and mapping of the cerebral blood vessels at the same level using either staining with DiI (**b**) or India ink (**c**,**d**). (**c**,**d**) Black staining: India ink; blue counter staining: toluidine blue. Between the two techniques for vasculature staining, DiI is more sensitive. However, India ink more clearly shows the typical curved vascular staining observed in the choroid plexus, which is prominently displayed in the fUS image. (**d**) High-power magnification of **c**. Functional ultrasound (fUS) imaging evoked in the left (**e**) or right (**f**) somatosensory cortex, hindlimb part (S1HL) using electrical stimulation (5 Hz, 0.2 mA, 100 μs width for 10 s, separated by 20 s) of the right (**e**) or left (**f**) sciatic nerve applied either on the right (**e**) or left (**f**) side, respectively. (**g**) Time course changes in the evoked fUS response in the left S1HL following stimulation of the contralateral sciatic nerve. Black bar: duration of the stimulation. Electrical stimulation of the left hindpaw induced a reproducibly increased fUS signal in the contralateral (right) S1HL. Scale bars, 2.3 mm (**a**,**b**), 1.5 mm (**c**), 375 μm (**d**), 1 mm (**c**–**f**).

We have previously shown that fUS allows measurement of functional haemodynamic changes induced by sensory stimulation^17^. To test whether fUS is capable of detecting correlated patterns, we aimed to measure spontaneous haemodynamic fluctuations between contralateral homologous cortical areas that share similar function and are massively interconnected by axonal projections through midline commissural structures such as the corpus callosum^21^. To achieve this, we first identified functionally correlated contralateral cortical areas activated by electrical stimulation of the right or left sciatic nerve. Preliminary mapping of the rat brain vasculature (Fig. 1a–d) allowed recognition of the blood vessel architecture of large-scale brain regions on the coronal level where the fUS imaging was performed. Figure 1e,f show the vascularization of the rat brain detected with Ultrafast Doppler imaging, over which we superimposed the evoked fUS signal, measured using the fUS sequence number 1. We have denoted regions that correspond to the rat brain atlas of Paxinos^22^. Accordingly, five consecutive electrical stimulations induced a robust and reproducible increase in haemodynamic response in the contralateral primary sensory cortex, hindlimb part (S1HL) and primary motor cortex (M1) regions (Fig. 1g).

### fUS detection of FC

**Figure 2:**
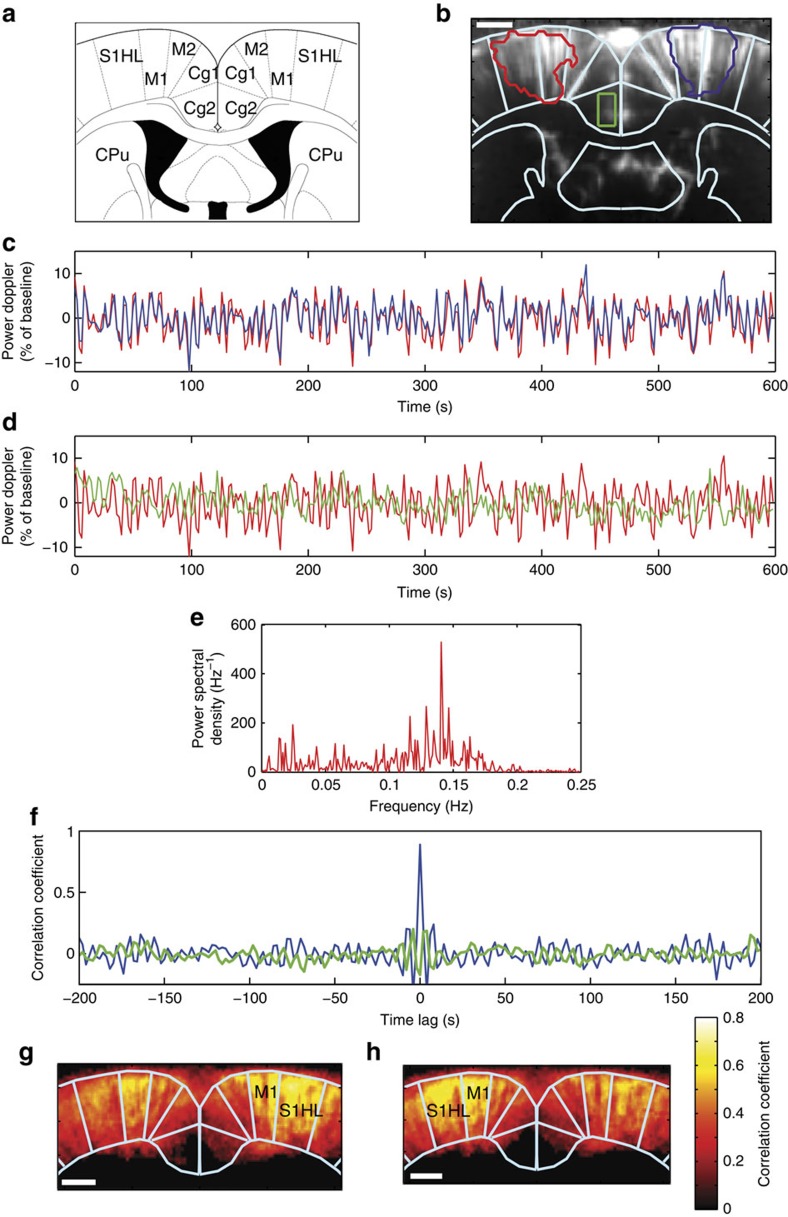
Spontaneous haemodynamic signal variations show high temporal correlation in contralateral S1HL+M1 regions. (**a**,**b**) Previously determined seed regions, that is, the right (blue) and left (red) S1HL+M1. (**c**) Spontaneous variations in the power Doppler signal in the seed regions marked by the evoked blood flow response of the right (blue curve) and left (red curve) S1HL show high temporal correlations. The curves shown are typical in terms of fluctuation amplitude. (**d**) Example of temporal variations in signals that are weakly correlated in the S1HL+M1 and the ipsilateral secondary cingulate cortex. (**e**) Frequency distribution of the power spectral density in the seed regions (S1HL+M1, in red in **b**). (**f**) Temporal correlation function between the signals obtained at the seed region (left S1HL+M1, red delimitation in **b**) and the contralateral S1HL+M1 region (blue curve, corresponding to the zone delimited in blue in **b**) or between the same seed region and the ipsilateral secondary cingulate cortex (green curve, corresponding to the zone delimited in green in **b**). Averaged spatial pattern of cortical regions temporally correlated to the right (**g**) or left (**h**) S1HL+M1 seed regions (*N*=6). Scale bars, 1.1 mm (**b**), 1 mm (**g**,**h**).

**Figure 3:**
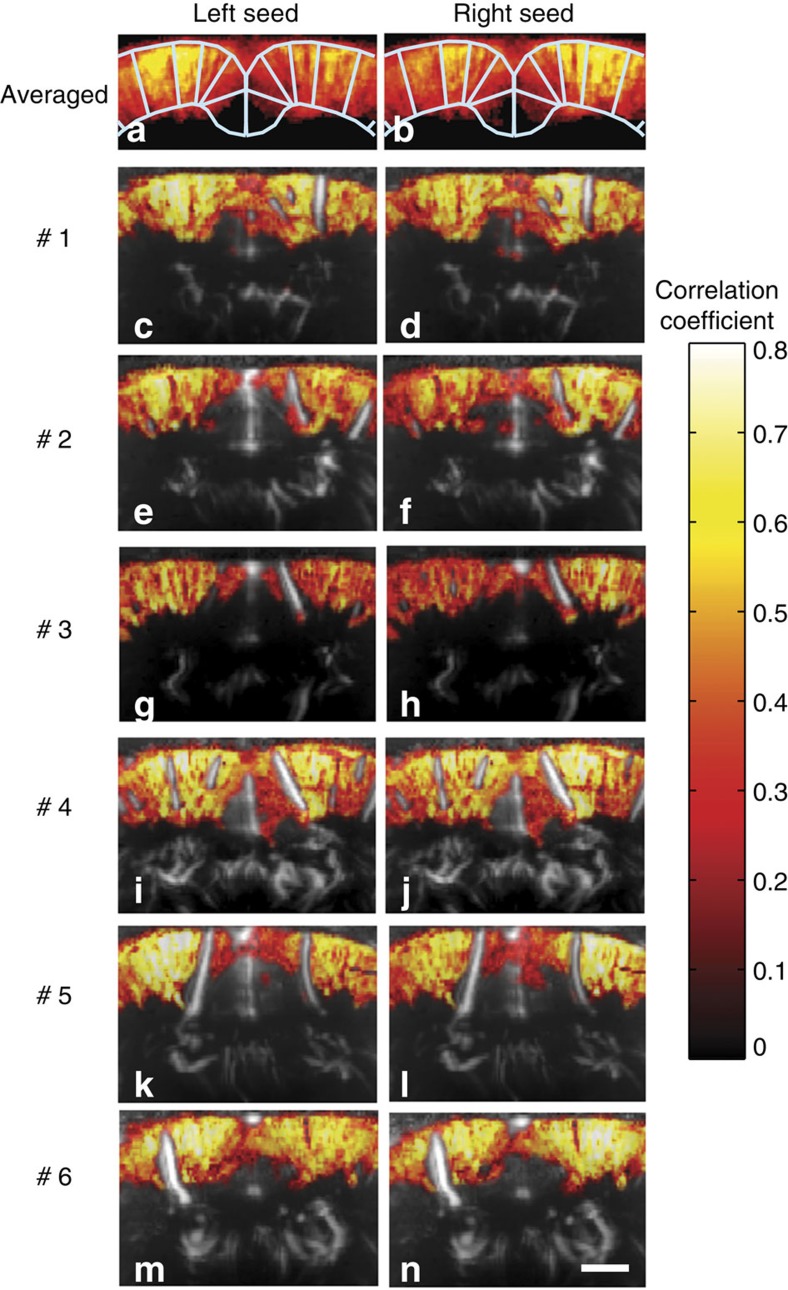
Inter-animal variability in the spatial pattern of temporally correlated spontaneous variations of the haemodynamic signals. Correlations in the haemodynamic signal measured in the previously identified left or right S1HL+M1 seed regions. (**a**,**b**) Averaged from six animals. (**c**–**n**) Individual results in the six different rats. Results are expressed as the percentage of correlation (100%=1), colour coded and superimposed on the individual haemodynamic map of the respective animal. Scale bar, 1.6 mm.

Next, we asked whether functionally and anatomically connected regions show correlated changes in spontaneous fUS signal fluctuations. Once the bilateral S1HL and M1 regions were functionally identified (Fig. 2a,b), the spontaneous fUS signal of this coronal slice was measured, without stimulation, for 10 min. Figure 2c compares the temporal variation of the spontaneous power Doppler signals of the two ‘seed regions’, respectively, identified based on the previously evoked blood flow response of the right (blue delimitation) and left (red delimitation) sciatic nerve stimulations (Fig. 2b). Strikingly, the fUS signals from these two contralateral, but functionally similar regions were highly correlated (correlation coefficient *r*>0.8). The correlation function (blue curve in Fig. 2f) also indicated that the two signals were in phase, as the maximum of the correlation function occured at a zero lag time point. Notably, not all cortical regions were similarly correlated. Figure 2d compares the signal from the left seed (red delimitation in Fig. 2b) to a signal extracted from the left anterior secondary cingular cortex (green delimitation in Fig. 2b). These signals were less correlated than the previously analysed contralateral regions (correlation coefficient *r=*0.25). The low-amplitude fluctuations of both correlation functions in Fig. 2f display no secondary maximum, indicating that the fluctuations of the different time signals were random. Analysis of the power spectral density of the signal of the left seed (red delimitation in Fig. 2b) revealed that its temporal variations were of low frequency (90% of the total power spectral density is below 0.15 Hz, Fig. 2e). Mapping the cortical regions where the signal had an elevated correlation coefficient with the left or right S1HL and M1 seed regions enabled labelling of the bilateral, motor and sensory cortical areas (Fig. 2g,h) with high inter-animal reproducibility (*N*=6, Fig. 3). The mean Pearson correlation coefficient for both right and left ‘seed-based’ FC maps was 0.65±0.04 (*P*<0.001, *N*=6).

In conclusion, our results demonstrated that the fUS-measured, spontaneous low-frequency fluctuations of the CBF were highly correlated in the sensorimotor system. The frequency range and frequency distribution of the correlated spontaneous CBF fluctuations are in accordance with values reported by previous BOLD-based studies^1^,^10^,^23^,^24^.

### Seed-based determination of FC at different coronal planes

**Figure 4:**
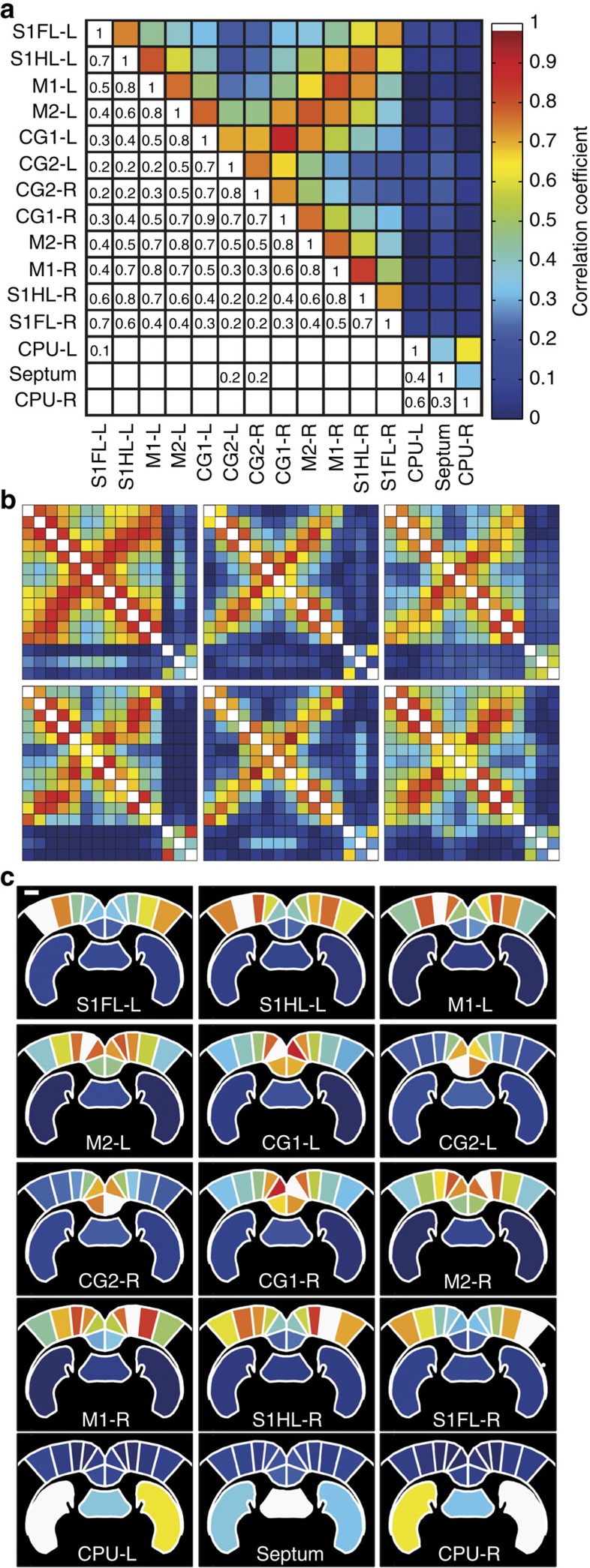
fUS-detected functional connectivity measured at Bregma −0.6 mm by using the ‘seed-based’ approach. The averaged (**a**) and individual (**b**) correlation matrices in six animals and the projection of the averaged correlation values on a schematic representation of the brain areas (**c**), with the seed region indicated in white. These results show that fUS is able to reproducibly measure temporal correlations in the spontaneous low-frequency fluctuations of the CBF. These correlated fluctuations indicate functional connectivity, observed principally between the contralateral cortical areas and somatosensory and motor areas. Scale bar, 1mm.

**Figure 5:**
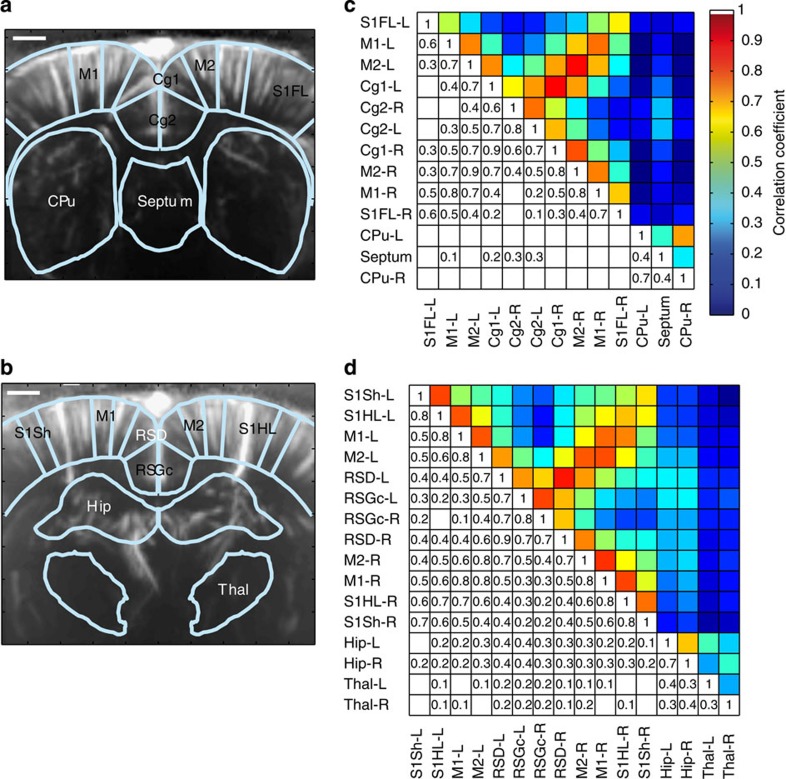
Correlation matrices of the functional connectivity. (**a**,**c**) Matrices at Bregma +0.84 mm and (**b**,**d**) matrices at Bregma −2.16 mm. (**c**,**d**) The correlation matrices obtained show a strong bilateral correlation of signals in the cingular, retrosplenial granular, motor and S1 cortices, as well as between the motor and S1 cortices. Other brain areas, such as the caudate putamen, septum, hippocampus and thalamus showed bilateral correlations and weak-to-no correlation with the somatomotor areas. Scale bar, 1.1mm.

In addition to the coherent activity of the sensorimotor system reported above, BOLD-based studies have reported patterns of intrinsic FC in many other neuroanatomical systems, including visual^25^, auditory^26^, hippocampal^26^, dorsal attention^25^ and ventral attention systems^5^,^6^,^25^. To investigate whether coherent fUS measurements would reveal similar spatial patterns, we assayed the FC in three different coronal planes, based on calculation of the mean Pearson correlation factor in the fUS signal between the anatomically defined regions of interest (ROI). These ROIs were obtained through overlaying the power Doppler vascular map with the spatial referential frame of the Paxinos atlas^22^. The results are presented either as correlation matrices (Fig. 4a,b) or as the projection of these correlation factors on the schematic atlas view (Fig. 4c). The correlation coefficient matrices were highly reproducible between individual animals. The mean Pearson correlation coefficients of the FC matrix intercorrelation coefficient, using only the non-diagonal values, were 0.85±0.03 (*P*<0.001, *N*=6) at Bregma −0.6 mm, 0.86±0.04 (*P*<0.001, *N*=4) at Bregma +0.84 mm and 0.86±0.04 (*P*<0.001, *N*=5) at Bregma −2.16 mm. The mean correlation matrix of each coronal plane is displayed in Figs 4a and 5c,d. All of the matrix coefficients that were >0.2 were reproducible (*P*<0.05). Only the reproducible coefficient values (*P*<0.05) are displayed in these figures.

**Figure 6:**
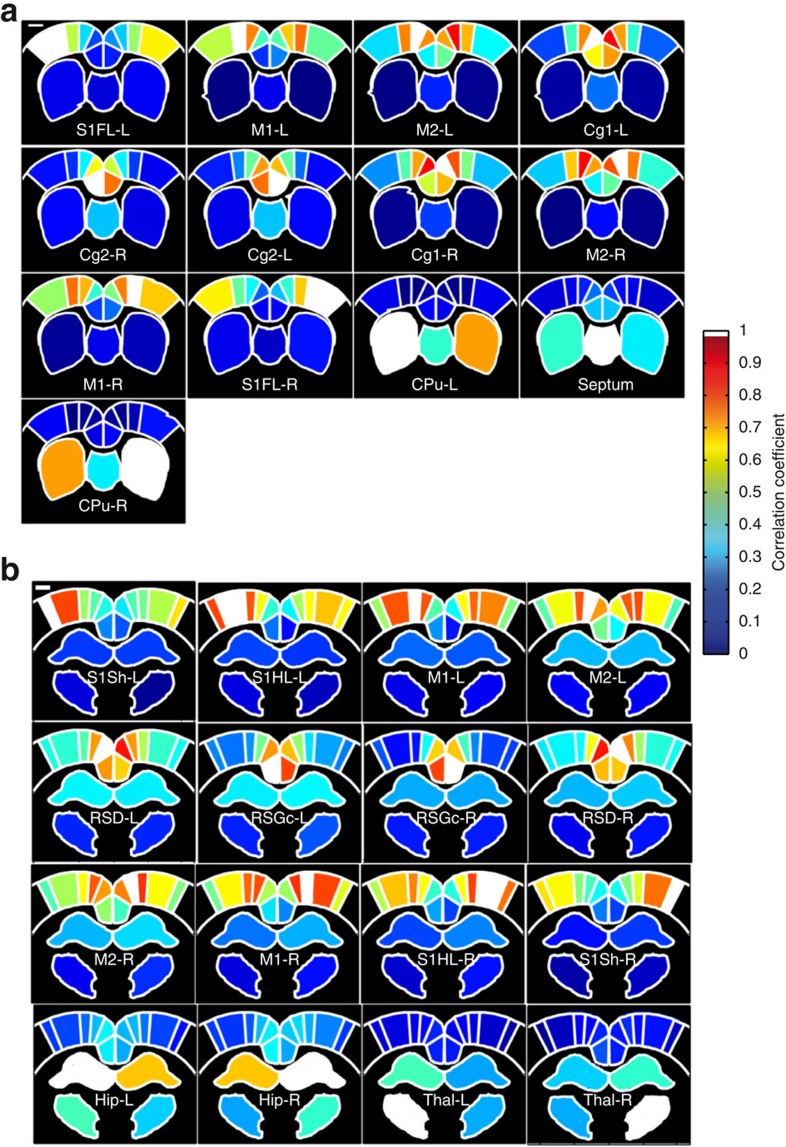
Spatial representation of temporally correlated fUS signals. Correlation coefficients of the temporally correlated fUS signals measured at Bregma +0.84 mm (**a**) and Bregma −2.16 mm (**b**), and presented in correlation matrices on Fig. 5, with the seed region indicated in white. Scale bar, 1mm.

Overall, the spatial patterns of the coherent fUS activity showed strong bilateral correlations in functionally heterogeneous brain areas, such as the caudate nucleus and putamen (CPu, *r*=0.6±0.1, *P*<0.001, *N*=6, Fig. 4a and *r*=0.72±0.08, *P*<0.001, *N*=4, Fig. 5c, respectively) and the hippocampus (*r*=0.7±0.1, *P*<0.001, *N*=5, Figs 5d and 6b). These correlations were also reported in areas previously described as the ‘sensory-motor resting-state network’^24^,^27^ (coefficient correlation *r*=0.6–0.9), that is, the primary and secondary cingulate cortex (Fig. 4a), retrosplenial granular and retrosplenial dysgranular cortex (Figs 5d and 6b), primary and secondary motor cortex (Figs 4a, 5c,d and ), and parts of the primary sensory cortex: S1HL or primary sensory cortex, forelimb part (S1FL) (Figs 4a, 5c,d and 6a).

Furthermore, the brain areas of the ‘sensory-motor resting-state network’ were overall highly connected to each other (for instance, the S1HL with the motor cortex, Fig. 4a), as previously described^27^. However, the cingulate cortex (both primary and secondary areas) was not correlated with other cortical structures, except the neighbouring primary motor cortex (Fig. 4a), as previously described^28^. By contrast, cerebral structures such as the CPu, septum, thalamus and hippocampus showed no correlation with the ‘sensory-motor resting-state network’ (Figs 4a,c and 5c) and little or no correlation with each other. These observations are consistent with the previous description of their respective implications in segregated pallidum-like (septum), thalamic (thalamus) and retrohippocampal (hippocampus) resting-state networks^27^.

In addition to reproducing the spatial patterns previously reported by fMRI BOLD imaging, the high sensitivity and high spatial resolution (100 μm × 100 μm in-plane pixel size) of fUS imaging allowed the identification of additional specific connectivity patterns between parts of the above brain areas, such as the preferential connection of the cingulate cortex with the primary, but not secondary, motor cortex and a strong connection between different parts of the primary sensory cortex, such as the S1HL and S1FL (Fig. 4a,c). In conclusion, fUS imaging was able to detect FC patterns of distinct neuro-anatomical systems with 100 μm spatial resolution.

### Filtering of high-frequency physiological noise

**Figure 7:**
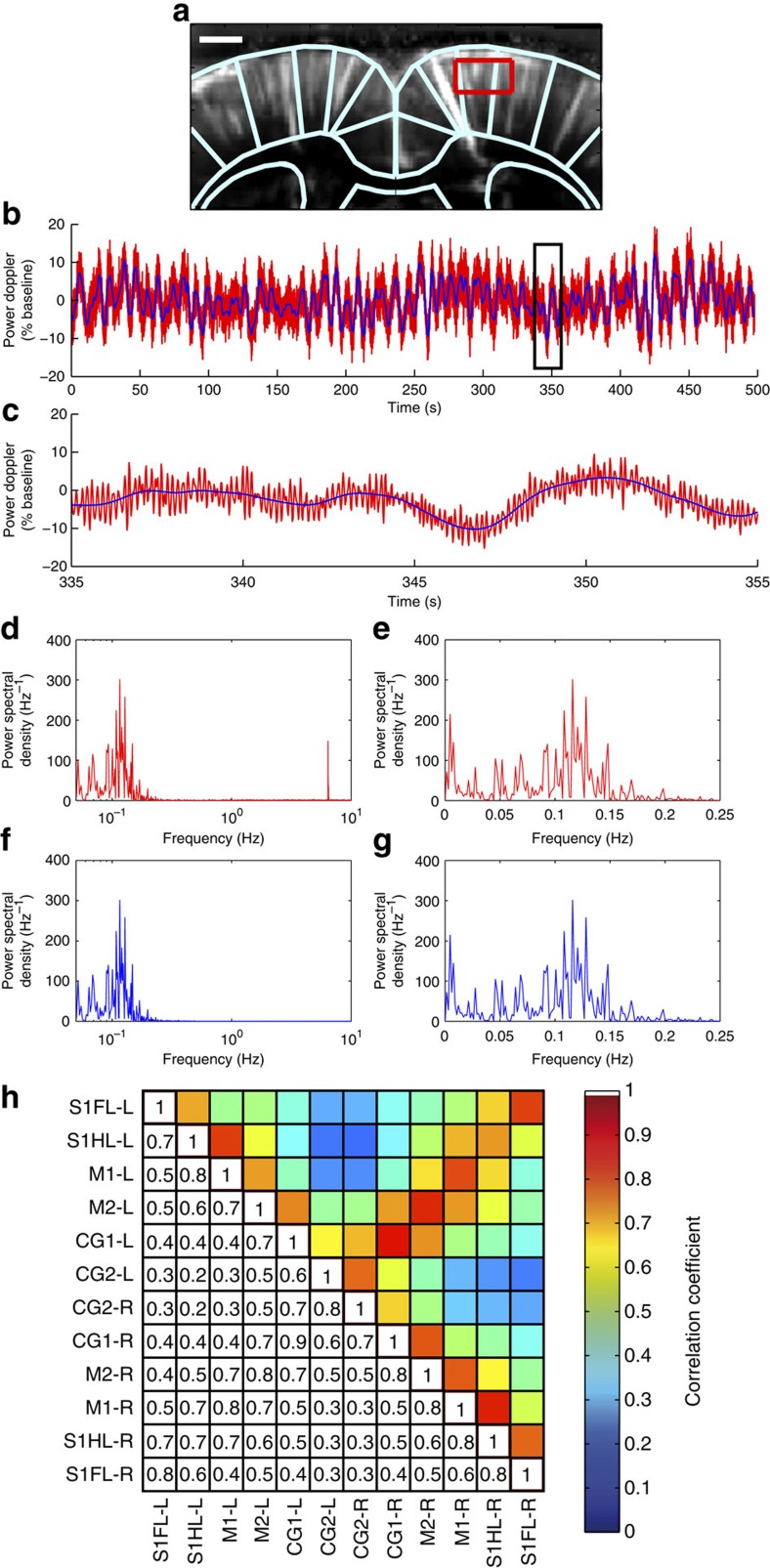
High-frequency physiological noise can be completely removed from the spontaneous fUS signal. (**a**) The area boxed in red on the vascular map was analysed using the continuous high frame rate fUS sequence number 2. Scale bar, 1 mm. (**b**) fUS signals obtained from the area labelled on **a**, with (blue) and without (red) low-pass (butter third order, 0.5 Hz frequency cutoff) filtering of cardiac pulsatility. (**c**) Expansion of the area boxed in **b** reveals oscillations resulting from cardiac pulsatility (red), superimposed on low-frequency spontaneous variations of the fUS signal (blue). (**d**) Power spectral density of the raw signal obtained from the area labelled in **a** before cardiac pulsatility filtering (semilog scale). The low-frequency peak (~0.1 Hz), zoomed in **e** (linear scale), represents the functional connectivity signal. The high-frequency peak (~7 Hz) is the biological noise due to the cardiac pulsatility. (**f**,**g**) Power spectral density of the signal obtained from the area labelled in **a** after cardiac pulsatility filtering. The low-frequency components of the functional connectivity (**g**) are not disturbed by the filtering. (**b**–**g**) Representative results obtained from one animal. (**h**) Mean functional connectivity matrix of dorsal cortical regions at Bregma +0.8 mm obtained with the continuous high frame rate fUS sequence number 2 followed by pulsatility filtering (in *N*=5 animals). This matrix is highly similar to the connectivity matrix obtained using the intermittent frame rate, low-bandwidth sampling sequence number 1, shown previously in Fig. 4a. The mean Pearson correlation coefficient of the FC matrix intercorrelation coefficient, using only the non-diagonal values, was 0.80±0.05, *P*<0.001, *n*=5.

The cerebral blood volume fluctuates with the cardiac cycle (around 5 Hz), which could lead to biased measurements of the low-frequency components, a well-known concern for fMRI BOLD imaging^29^,^30^. Therefore, to precisely evaluate the components of the spontaneous fUS signal fluctuation patterns, we continuously measured the fUS signal in the neocortex for 500 s at our standard 500 Hz sample rate (fUS sequence number 2). This high frame rate, which was chosen to adequately sample the Doppler ultrasound signal without aliasing^31^, oversamples the fUS variations resulting from both intrinsic FC and cardiac pulsatility. Indeed, as shown by the red curve in Fig. 7b,c, which was computed from the area boxed in red in Fig. 7a, both low- and high-frequency fluctuations were observed. The power spectral density revealed two peaks: one low frequency (<1 Hz) and a second one at ~7 Hz (Fig. 7d,e), which corresponded to the pulsatility of the CBF. The low-frequency components were grouped around 0.1 Hz (Fig. 7e), the frequency range of the intrinsic FC^1^. This demonstrates that the blood pulsatility signal is not negligible compared with the intrinsic FC signal.

As the pulsatility was correctly sampled with fUS sequence number 2, accurate filtering of the pulsatility could be performed. Indeed, low-pass filtering (butter third order, 0.5 Hz frequency cutoff) resulted in the complete removal of the high-frequency fluctuations (Fig. 7f), without changing the low-frequency components (Fig. 7g), generating the blue curve in Fig. 7b,c.

Because of the practical limitations in computing bandwidth (see Methods), the continuous imaging sequence number 2 is currently not adapted for real-time analysis of large brain areas. However, the cardiac cycle was already fully sampled with the intermittent (2 s) low-bandwidth sampling sequence number 1 (500 Hz frame rate with a sampling time of 400 ms, corresponding to two cardiac cycles). Therefore, the mean value of this signal acted as an efficient low-pass filter that removed most of the pulsatility signal. Accordingly, the results obtained by using the sequence number 2 were fully consistent with the results given by sequence number 1 (Fig. 7h). The Pearson correlation coefficient computed between the two FC matrices using only the non-diagonal coefficients of the two different acquisition types was *r*=0.97, establishing that the intermittent sequential acquisition sequence number 2 was not biased with pulsatility artefacts.

In conclusion, both of the fUS acquisition sequences efficiently filtered out the cardiac pulsatility noise. Consequently, all coherent spatial patterns of intrinsic connectivity were based on signals of low-frequency changes specific to the modulation of CBF.

### Data-based identification of anticorrelated FC patterns

**Figure 8:**
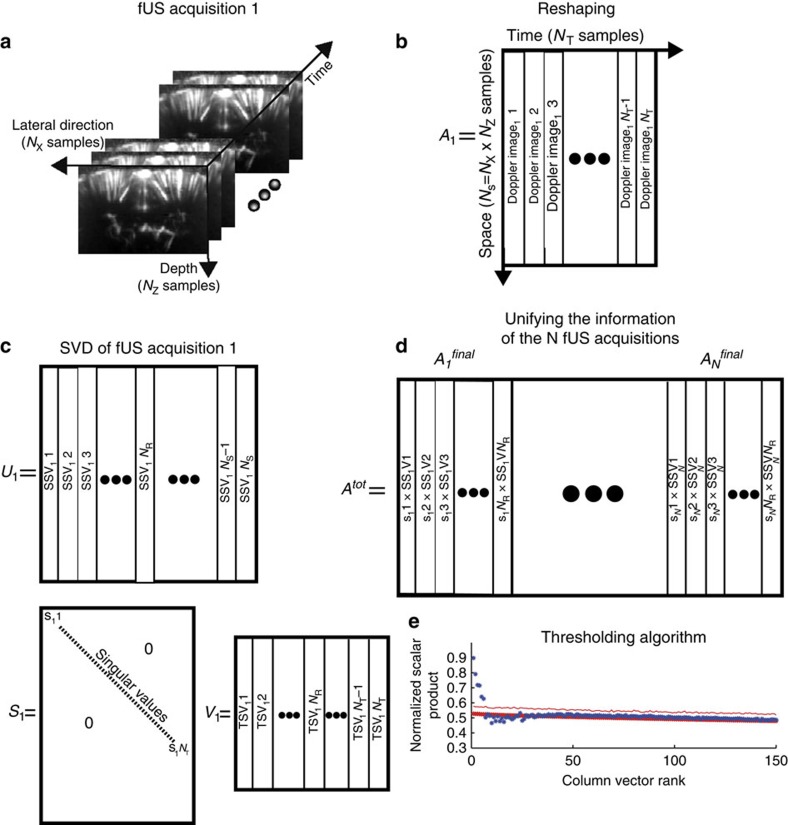
Singular value decomposition processing. (**a**) Description of the fUS acquisition as a three-dimensional matrix. (**b**) Reshaping the fUS three-dimensional acquisition matrix into a two-dimensional matrix for singular value decomposition. (**c**) Result of the singular value decomposition of a fUS matrix. (**d**) Combination of the information from the N fUS acquisitions. (**e**) Results from the thresholding. Blue dots: values of the GSMs with the noise removed. Red dots: simulated Gaussian random noise. Plain line: 2 × the s.d. of mean noise. The GSMs with a value higher than this level were considered significantly different from noise.

Interestingly, BOLD-based connectivity studies have suggested that regions with apparently opposing functionality display temporally anticorrelated spontaneous signals, both in human and rodent brains^28^,^32^,^33^. Specifically, externally focused or task-related networks are anticorrelated with the intrinsic default-mode network^28^,^32^. In BOLD-based studies, pre-processing removal of both neuronal and non-neuronal global noise is often required to visualize these patterns, but the potential introduction of artefactual coherence patterns is a subject of ongoing debate^30^,^34^,^35^,^36^. As fUS-based measurements of intrinsic connectivity are free of high-frequency physiological noise (see above), we investigated in detail the connectivity patterns in rodent brains, without global noise extraction, to reveal these complex intersystem relationships. For this purpose, we used singular value decomposition (SVD) as a linear decomposition operator, which provided powerful data decomposition without any spatial or temporal information *a priori.* After SVD-based noise removal (see Methods), we identified the global spatial modes (GSMs) of the SVD decomposition, which represent the mean (that is, global) FC for the entire population of *N* animals at each of the three investigated coronal planes (Fig. 8).

**Figure 9:**
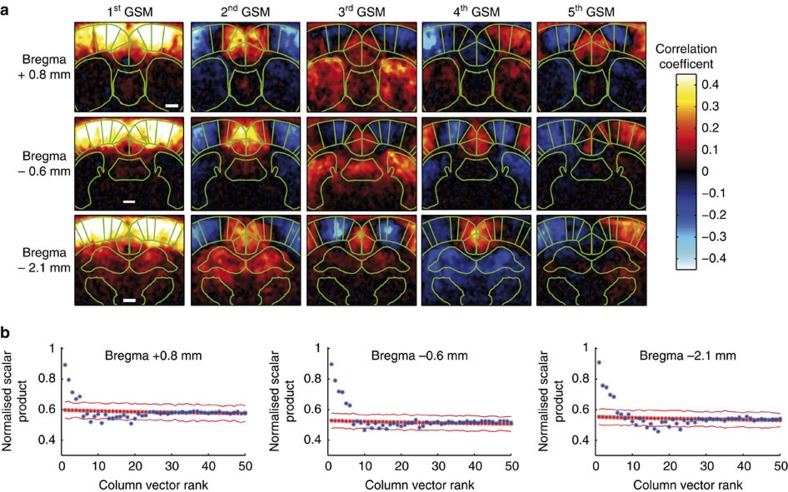
Anticorrelated fUS connectivity patterns reveal distinct functional networks at three different coronal planes. (**a**) Global spatial modes (GSMs), representing reproducible connectivity patterns (*N*=6), at the three investigated coronal levels. The first GSMs show synchronized haemodynamic fluctuations in the cortical ribbon, which did not include the secondary anterior cingulate cortex (see Figs 2 and 5 for anatomical definitions). Subsequent GSMs delineate highly contrasting cortical connectivity patterns, where the midline anterior cingulate and retrosplenial cortices (red and yellow on GSM 2 and 3), prominent hubs of the putative default-mode network are temporally anticorrelated with the task-dependent lateral sensorimotor network (blue and light blue on GSM 2 and 3, respectively). These two main cortical networks are also correlated with subcortical regions such as the caudate, putamen and septum. Scale bar, 1 mm. (**b**) Results of the thresholding procedure, as described in Methods. Blue dots: values from the GSMs with noise removed. Red dots: simulated Gaussian random noise. Plain red line: 2 × the s.d. of mean noise. GSMs with a value higher than this level were considered significantly different from the noise.

At least five or six GSMs were above the noise level at each coronal plane (at Bregma +0.84, +0.6 and −2.16 mm), reproducibly revealing highly contrasted FC networks (Fig. 9a,b). The first GSM, corresponding to the most prominent connectivity pattern at each coronal plane, was dominated by cortical regions including the sensory, motor, frontal, cingulate and retrosplenial cortices (Fig. 9a), indicating strong, direct bilateral communication across the cortical ribbon^24^. Notably, this pattern did not include the secondary anterior cingulate cortex, a region that showed a strong correlation with the primary anterior cingulate cortex using seed-based analysis (Fig. 4a). Interestingly, the second most prominent connectivity pattern (GSM 2) at each coronal plane identified the midline-structure anterior cingulate and retrosplenial cortices, and the dorsal hippocampus at Bregma −2.16 mm. These structures were negatively correlated with the more lateral parts of the cortical ribbon of GSM 1 on all three coronal planes, with the exception of the most medial part of the secondary motor cortex (M2). The third GSM at the two most rostral planes revealed a positive correlation of the contralateral anterior cingulate cortices and the dorsal part of the CPu, and these networks were also negatively correlated with the more lateral motor and somatosensory cortices (Fig. 9a). Finally, the fifth GSM, at Bregma +0.84 mm, and the fourth GSM at Bregma +0.6 and −2.16 mm, showed a strong anticorrelation of the midline anterior cingulate and retrosplenial cortices with the more lateral motor cortices (M1 and M2), as well as a strong bilateral connection of the subcortical regions, such as the CPu at Bregma 0.84 and −0.6 mm (Fig. 9a), and the dorsal hippocampus and thalamus at Bregma −2.16 mm (Fig. 9a).

In conclusion, SVD analysis of fUS connectivity correlations separates, at high spatial resolution, several overlapping and previously unreported FC patterns that correspond to anatomically well-defined structural connectivity networks.

## Discussion

Coordinated spontaneous brain activity, indicating functional brain connectivity^1^, has received considerable attention in recent years. It has been investigated with multiple modalities, predominantly based on fMRI^1^,^37^, but also with other functional imaging techniques such as optical imaging^38^, positron emission tomography^3^, magnetoencephalography and electroencephalography^39^,^40^. The data obtained through these different modalities, each with its own specificity, sensitivity and spatiotemporal resolution, represent different types of physiological signals and are affected by different artefacts^41^. In this study, by using a large thinned-skull cranial window preparation and a novel ultrasonic sequence for ultrafast imaging of intrinsic FC of the living adult rat brain, we showed that fUS is highly efficient for mapping FC in a rodent model. This introduces a new neuroimaging modality for FC research with several novel key features.

First, we demonstrated that we were able to detect, both using seed-based and SVD analysis, strong and highly contrasting spatial coherence signals in low-frequency (<0.1 Hz) spontaneous fUS signal fluctuations. The resulting intrinsic FC patterns were similar to known major functional networks described in humans by using resting-state fMRI, such as the lateral task-dependent network putatively anticorrelated with the midline default-mode network. This type of anticorrelated activity is an important feature of dynamic brain organization^32^, putatively important for efficient cognitive function^42^,^43^, and has clinical predictive power in the treatment of depression^44^. However, preprocessing removal of global noise in fMRI-based studies potentially introduces artefactual coherence patterns and is a subject of ongoing debate^30^,^34^,^35^,^36^,^41^,^45^,^46^. Here the high temporal resolution of the fUS imaging coupled with the SVD analysis enabled us to show, without preprocessing removal of neuronal or non-neuronal global noise, the prominent presence of highly contrasting and anticorrelated connectivity patterns, which are similar to functional networks previously described in humans. Current developments are now underway to apply fUS to transcranial imaging in mice, through increasing the ultrasonic frequency range, to exploit the genetic tools available in this model organism.

Second, the reproducibility and robustness of this ultrasound method was demonstrated by comparing the seed- or SVD-based FC maps obtained from different animals. Notably, the mean Pearson intercorrelation coefficient of the seed-based FC matrix using only the non-diagonal values was at least 0.85±0.03 (at Bregma −0.6 mm) and was typically statistically significant (*P*<0.001) on all coronal planes. Similarly, for the three coronal planes investigated in this study, SVD analysis reproducibly identified at least five distinct mean GSMs (*N*=6) above the noise level.

Third, fUS imaging is able to spatially resolve changes in cerebral haemodynamics at 100 μm in-plane, which is higher than the typical 400 μm in-plane voxel dimension in recent fMRI studies of the rat brain^13^,^28^. This fourfold increase in spatial resolution considerably multiplies (4^2^=16-fold per slice) the amount of potentially available connectivity data. In addition to a more precise and detailed mapping of the FC networks, this may provide better imaging sensitivity at relatively small voxel volumes because of the elevated possibility of spatial filtering, a method that most investigators apply to improve the functional signal-to-noise ratio (SNR) of BOLD measurements^47^. In the near future, the extension of fUS to three-dimensional dynamic imaging using two-dimensional matrix array technology could lead to a 4^3^=64 times increase per volume in the number of available FC data compared with fMRI.

Fourth, the ultrafast 2 ms time resolution of fUS enables unambiguous discrimination between the resting-state activity and pulsatility artefacts before any SVD or independent component analysis (ICA) processing. This is important because the issue of cardiac and respiratory motion remains a potential concern in fMRI acquisitions^30^. Indeed, cardiac and respiratory cycles occur at around 6 and 1 Hz, respectively. Consequently, these signals become strongly aliased at typical repetition times (~1.7–5 s) in small animal fMRI, causing significant noise at frequencies typical of resting-state networks. Although De Luca *et al*.^29^ showed that probabilistic ICA can partly solve this issue, and Chang and Glover^46^ proposed a model-based noise-removal approach in human brain imaging, the usability of these approaches has not yet been demonstrated in rodent fMRI, where the temporal constraints for cardiac and respiratory motion are much higher.

How closely does the fUS signal reflect neuronal activity, and how does this compare with the sensitivity and fidelity of the BOLD signal, the gold standard method for FC studies? In the current models of functional brain imaging, neural activity produces complex local changes in the CBF, the cerebral metabolic rate of oxygen (CMRO_2_) and cerebral blood volume following task-activation and during resting state (recent review by Liu^48^). In the BOLD signal model, neural activation-induced fractional BOLD signal change is related to underlying changes in the CBF and CMRO_2_ (refs 49, 50). Because of the limited diffusibility of oxygen from the blood to the brain, the activation-induced increases in CBF largely overcompensate that of CMRO_2_. The difference between these two quantities yields a relatively moderate (compared with the CBF change in amplitude) reduction in the deoxyhaemoglobin content of the brain microvasculature, elevating the local magnetic resonance signal^49^,^50^. Consequently, the major parameter that determines the change in the BOLD signal, similarly to fUS, is the change in CBF. However, the amplitude of the BOLD signal (and consequently its SNR) is reduced by the parallel increase in CMRO_2_. Therefore, the direct measurement of local changes in CBF using fUS is a valid readout of localized neuronal activity, which is also a potentially more sensitive method than BOLD. In addition, by directly measuring the changes in the microvascular haemodynamics, fUS measurements are also free of another potential fMRI confound—blood CO_2_ concentration, which can change with the respiration rate, leading to BOLD signal changes that are unrelated to neural activity^30^.

Importantly, previous flow-sensitive MRI imaging in human subjects revealed coherent fluctuations in the CBF with similar spatial patterns to those seen with BOLD, both after task activation and during the resting state^51^,^52^,^53^,^54^,^55^. Based on these premises, the CBF was already proposed as a biologically more specific correlate of neural activity compared with the BOLD contrast image^56^,^57^,^58^, because of the more direct link between CBF and neuronal activity, as discussed above. Indeed, change in the CBF has the potential for a more accurate estimation of the location, magnitude and longitudinal variation of neural function, being more linearly related to changes in neural activity than BOLD^59^ and having decreased intersubject and intersession variability, which permit a smaller sample size for a given effect size^56^,^57^,^60^. However, current MRI-based measurements of CBF have significantly lower sensitivity and temporal resolution than BOLD^58^. The fUS approach presented in our study overcomes these limitations and provides a novel perspective in FC imaging through direct, sensitive and highly resolved measurements of a biologically well-defined physiological quantity, CBF.

In present, constraint to three-dimensional (two dimensions in space plus time) imaging is a limitation of this study compared with fMRI. The extension to four dimensions will require the use of matrix arrays coupled to ultrasonic platforms with a great number of channels embedded and adequate graphic processing unit computational power. Nevertheless, such exponential developments are envisioned in the near future. Of particular interest, the number of achievable raw data sets used in fUS should reach a 64-fold increase (typically 100 μm versus 400 μm) in space and 1,000-fold increase in time (typically 1 ms versus 1 s) compared with fMRI. This will lead to a fourfold increase in the available data compared with current state-of-the-art imaging modalities. The impact of this increase on the SNR and robustness of data processing algorithms for FC studies should be carefully studied in future works.

In comparison with fUS, optical techniques such as optical intrinsic signal imaging can reach higher spatial (~1 μm) and comparable temporal resolutions (1 ms) than fUS by using a similar thinned-skull approach on rats, but it remains limited to the cortex surface due to the elevated scattering of light in living tissue. To increase the imaging depth, two strategies were developed. First, approaches based on fNIRS (functional near-infrared spectroscopy) or diffuse optical imaging can be used to recover deeper brain images, however with significantly lower spatial resolution (>1 cm)^61^. Second, new multi-wave approaches such as photo-acoustics propose to solve this problem by taking benefit of the interaction between acoustic and optical waves, getting optical contrast and acoustical resolution in deep tissues. However, these approaches require the complex use of ultrasonic scanners coupled with laser sources^62^.

Compared with optical approaches, the major advantage of fUS consists in providing whole-brain functional imaging with a very good spatial and temporal resolution. Moreover, the fact that fUS relies solely on the use of ultrasonic waves at ultrafast frame rates enables to design very small imaging probes that can be implanted in the skull, paving the way to functional brain imaging of awake and freely moving animals. Finally, ultrafast ultrasound imaging (the core of resting-state fUS) can provide several other valuable information such as vascular resistivity^63^, tissue strain imaging and tissue pulsatility.

The thinned-skull imaging window size is also a putative limitation that impeded, in the present study, the imaging of laterally located areas, such as the amygdala, rostral structures such as the olfactory bulb, and caudal structures such as the brain stem. However, as deeper lying brain structures, such as the ventral parts of the CPu (see Fig. 8a), were well resolved, the experimental protocols may be easily adapted for fUS imaging of almost all lateral, rostral and caudal brain areas.

The prospect of resting-state fUS are exciting because the current development of light, portable ultrasonic probes will soon allow FC mapping in awake and freely moving animals, without motion artefacts that may perturb fMRI studies, even in human subjects^30^. Although recent studies have shown that fMRI-based FC imaging is possible in restrained, awake rats after training^24^,^64^, important experimental protocols, such as the task-related deactivation protocol that is cardinal for the definition of the default-mode network^65^, have not yet been realized because of the requirement for tight physical restraint during BOLD imaging.

Finally, the translation of resting-state fUS imaging to clinical applications is very promising. Although passing through the skull bone with ultrasonic beams remains a challenge, two clinical applications of major interest are not limited by this restriction and are currently under development. First, non-invasive fUS imaging can be performed straightforwardly in newborns through the fontanel or the temporal window. Second, fUS imaging can be performed peroperatively during surgery. These clinical applications are of major interest, as the use of fMRI is extremely difficult in these practical configurations. Consequently, resting-state fUS imaging may become complementary to resting-state fMRI. In particular, the ability of fUS to perform functional imaging of newborn brain activity with a portable bedside technology is a major advantage compared with fMRI. Therefore, recent ultrafast Doppler mapping of cerebral vasculature in neonates paves the way to resting-state fUS in paediatrics^63^.

After more than 25 years of functional neuroimaging, fMRI, positron emission tomography and electroencephalography/magnetoencephalography are well-established techniques. Our study demonstrates that fUS is a novel, additional neuroimaging modality, which enables FC mapping in rodents with unprecedented resolution and sensitivity.

## Methods

### Animals

All experiments were performed in agreement with the European Community Council Directive of 22 September 2010 (010/63/UE) and the local ethics committee (*Comité d’éthique en matière d’expérimentation animale* number *59, C2EA—59, ‘Paris Centre et Sud’*). Accordingly, the number of animals in our study was kept to the necessary minimum. Experiments were performed on 20 male Sprague–Dawley rats (Janvier Labs; Le Genest St Isle, France), weighing 200–225 g at the beginning of the experiments. Animals (three per cage) arrived in the laboratory 1 week before the beginning of the experiment. They were kept at a constant temperature of 22 °C, with a 12-h alternating light/dark cycle. Food and water were available *ad libitum*.

### Preparation of the large thinned-skull imaging window

To perform ultrasound imaging through the skull of adult rats, the skull was thinned to 50–100 μm over an area of ~1.2 cm × 0.9 cm at 1–7 days before imaging. This window dimension allows to use the central 128 elements of the transducer (0.08 mm per element × 128=10.24 cm large). Under anaesthesia (intraperitoneal (IP) injection of medetomidine (Domitor, 0.3 mg kg^−1^) and ketamine (Imalgène, 40 mg kg^−1^)), the head of the animal was placed in a stereotaxic frame and the three layers of bone were consecutively removed by drilling (Foredom, USA) at low speed using a micro drill steel burr (Fine Science Tools, catalogue number 19007-07). During the thinning procedure, which typically lasted 90 min per rat for a skilled experimenter, the skull was frequently cooled with saline and an airstream, as suggested previously^66^, resulting in a lack of heating, swelling or oedema of the cerebral cortex. The thinned window was protected by a small (1 cm × 1 cm) plastic cover and the skin was sutured using 5.0 non-absorbable Ethicon thread. Preliminary experiments showed that this method enabled good quality ultrasound imaging for as long as 1 week after skull thinning. The optimal results were obtained 24 h to 3 days after the preparation, as the bone tends to re-grow and the size of the ultrasound transparent window reduces with time, especially on the lateral sides.

### Animal preparation for ultrasound imaging

Animals were anaesthetized using an initial IP injection of medetomidine (Domitor, 0.3 mg kg^−1^) and ketamine (Imalgène, 40 mg kg^−1^), followed by hourly IP injections of medetomidine (0.1 mg kg^−1^ h^−1^) and ketamine (12.5 mg kg^−1^ h^−1^). Imaging sessions lasted 4–6 h. This dose was chosen according to previously published data on the observation of resting-state FC in rats^67^,^68^. In addition, we performed a preliminary experiment where we compared the use of medetomidine alone versus a combination of medetomidine and ketamine. We measured stable heart and respiratory rates, similar stimulation-evoked haemodynamic responses and intrinsic connectivity patterns with both methods. However, to minimize the possible pain and discomfort that the animal may be subjected to during the electrical stimulations of the sciatic nerve, we chose a mix of medetomidine and ketamine because of the analgesic properties of the ketamine. We observed that the animals needed to be under this anaesthesia for 2 h before the acquisition of intrinsic connectivity patterns to have stable and reproducible results.

For fUS imaging, the animals were placed in a stereotaxic frame (Stoelting; Chicago, IL, USA). Body temperature was maintained constant using a heating pad (Gaymar Industries, New York, NY, USA). For animals that received electrical stimulations of the sciatic nerve, a 1-cm incision was made in the skin and muscle of both hindpaws, above the femur. The sciatic nerve was exposed and isolated. A hook stimulating electrode was gently inserted to stimulate the sciatic nerve. The nerve was allowed to rest for 10 min before the beginning of the stimulation. Trains of five C fibre stimulations (5 Hz, 0.2 mA and 100 μs width for 10 s), separated by 20 s, were performed on each sciatic nerve, with coupled fUS imaging.

### Staining of the cerebral vascular architecture

Ten naive Sprague–Dawley rats were anaesthetized by IP injection of sodium pentobarbital (80 mg kg^−1^). Once the animal was deeply anaesthetized, a thoracotomy was quickly performed and an incision was made in the right atrium. For DiI staining (*N*=5 rats), 2 ml of saline, followed by 15 ml of DiI^69^ and then 10 ml of 4% paraformaldehyde were perfused intracardially at a rate of 7 ml min^−1^. The brain was removed and fixed for 2 days in 4% paraformaldehyde. Alternatively, (for *N*=5 rats), 5 ml of India ink was injected intracardially in a 1-min bolus. The brain was then immediately removed and fixed for 1 month in 4% paraformaldehyde. The brains were cryoprotected in 30% sucrose for two days. They were then frozen in cooled isopentane and sectioned (50 μm sections) using a cryostat (Microm Microtech France, Rhône, France). Free-floating sections were collected in 0.02 M phosphate buffer. Half of the sections were immediately mounted on Superfrost slides and mosaic images were acquired using a × 5 objective with an Axio Imager M1 microscope (Zeiss, Jena, Germany). The other half of the sections were mounted on Superfrost slides, air dried and counter-stained with 5% toluidine blue for 2 min. Finally, after two washes in distilled water, sections were dehydrated for 2 × 5 min in 50% ethanol, 2 × 5 min in 70% ethanol, 2 × 5 min in absolute ethanol and 2 × 5 min in xylene. Once dehydrated, coverslips were mounted with DPX (Sigma Aldrich, St Louis, MO, USA).

### Imaging the rat brain with fUS

Two hours after the induction of anaesthesia, the sutures and protective plastic cover were removed. The thinned skull was rinsed with sterile saline and 1 cm^3^ of ultrasound coupling gel was placed on the window. The linear ultrasound probe (15 MHz central frequency, 160 elements; Vermon, Tours, France) was positioned directly above the cranial window surface. We used the central 128 elements of the transducer, which was connected to an ultrafast ultrasound scanner (Aixplorer, SuperSonic Imagine, Aix-en-Provence, France). The software-based architecture of the scanner enabled Matlab (MathWorks, Natick, Massachusetts, USA) programming of custom transmit/receive ultrasound sequences. At the beginning of each session, an anteroposterior Doppler scan was performed to visualize and localize the shape of the blood vessels and the corresponding anteroposterior coordinates. Acquisitions were performed at three anteroposterior coordinates: Bregma +0.84, −0.6 and −2.16 mm. Note that ‘+’ (positive) signs mean ‘in front of’ (or ‘rostral to’) the Bregma, while ‘−’ (negative) signs mean ‘behind’ (or ‘caudal to’) the Bregma, corresponding to the rat brain atlas of Paxinos^22^.

### Ultrasound sequences

The concept of ultrafast Doppler relies on compounded plane-wave transmissions^70^. The brain was insonified with a succession of ultrasound plane waves. The backscattered echoes were recorded and beamformed to produce an echographic image for each transmission. The frame rate of ultrafast ultrasound can reach more than 10 kHz. However, a 500-Hz frame rate allows correct sampling of the ultrasound signals backscattered by the red blood cells without aliasing, in the rat brain, as demonstrated inref. 31. To increase the SNR of each echographic image taken at 500 Hz, we compounded the echographic images by transmitting several tilted plane waves and added their backscattered echoes. This compounded sequence resulted in enhanced echographic images, increasing the sensitivity of the Doppler measurement. In this study, the ultrasound sequence consisted of transmitting five different tilted plane waves (−4°, −2°, 0°, +2° and +4° tilted angle) with a 2,500-Hz pulse repetition frequency (PRF). The backscattered echoes were added to produce enhanced echographic images at a 500-Hz frame rate.

The current technological limit of the ultrafast imaging system is the high rate of recorded raw data (2 GB s^−1^), which cannot be stored in the computer memory and has to be treated in real time. This treatment consists of a beamforming step (image formation), which is time consuming. Therefore, we decided to perform two sequences:

Sequence number 1 consisted of insonifying the rat brain for 400 ms (2 cardiac cycles, 5 angles and a 2,500-Hz PRF), every 2 s, with sufficient spare time between acquisitions for in-depth beamforming of all the raw data. This allowed us to study the FC of both superficial and deep brain structures for acquisition periods lasting several minutes.

Sequence number 2 extended the temporal coverage of fUS acquisition by trading off spatial coverage. It consisted of in-depth insonification of the whole brain continuously for minutes at a 2,500-Hz PRF, but only beamforming in real time the dorsal part of the rat brain (the neocortex) because limited computer memory (6 hyperthreaded core processing unit and 24 GB random access memory). Indeed, limiting the region of interest to the neocortex resulted in 3 × less raw data and led to viable real-time processing with our current research platform.

### Power Doppler data treatment

The backscattered signals from the rat brain were composed of tissue and blood signals. As the blood moves faster than tissue, its signal frequency is higher and can be extracted by time filtering the data with a high-pass filter called clutter filtering (fourth order Butterworth filter with a 75-Hz frequency cutoff). For sequence number 1, as the acquisition was performed by blocks of 400 ms of insonification, the time fluctuations of the blood signal of each block were incoherently averaged to obtain a power Doppler image proportional to the cerebral blood volume. The sequence number 2 data treatment consisted of simply removing the tissue signal with the same high-pass filter.

### Building activation maps

Maps of ‘activated’ pixels were built using the Pearson correlation coefficient *r* between the local power Doppler temporal signal computed from each spatial pixel of the fUS acquisition and the temporal binary pattern induced by the electrical stimulations of the sciatic nerve. Activations were considered significant for a correlation *r*>2*σ*, where *σ* is the spatial s.d. of the correlation map. The time course for a given region was calculated by averaging the power Doppler signal over time for all pixels in the activated region (*r*>2*σ*).The amplitude of the power Doppler was represented as the percentage of change relative to the baseline in the activated region±s.d.

### Building FC maps

We employed the seed-voxel approach, which used the time course of the haemodynamic signal extracted from a ROI (the seed region), and determined the temporal correlation between its signal and the time course from all other brain voxels^10^. This created a whole-brain voxel-wise FC map of covariance with the seed region. This is a hypothesis-driven method, because it gives direct answers to specific hypotheses about the FC of the seed region. Such ‘seed-based’ FC maps correspond to maps of the Pearson’s product–moment correlation coefficient *r* between the time signal *α(t)*, which is the mean signal of the seed region and the power Doppler signal *s*_d_*(t)* for each pixel, defined as:


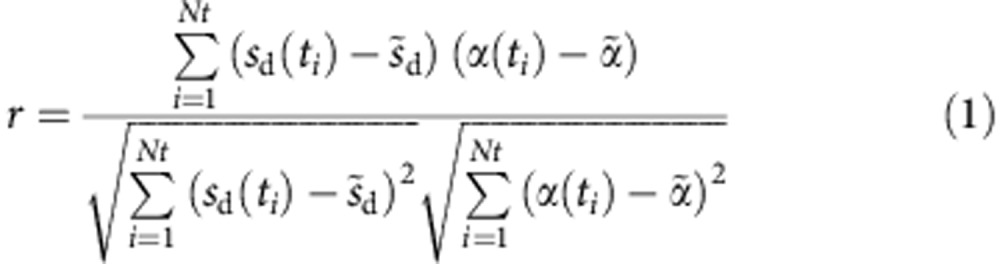


where ~designates the mean value of the variable over time. To superimpose ‘seed-based’ FC maps on the Doppler maps, we displayed only the pixels of the ‘seed-based’ FC maps in which coefficient *r*>2*σ*, where *σ* is the spatial s.d. of the correlation map. The reproducibility of the ‘seed-based’ FC maps between the different animals was determined without thresholding.

To increase the SNR, the time signal of each spatial pixel was filtered with a band-pass filter with cutoff frequencies of 0.05 and 0.2 Hz. The filtering of frequencies below 0.05 Hz was chosen to correct for a drift in the baseline, which could bias the correlation value. Filtering of frequencies over 0.2 Hz was chosen to remove noise, because we assumed that the frequencies of brain FC were below 0.2 Hz.

### SVD of the fUS data

Alternatively, data-driven analysis, such as SVD or ICA, can also be used to analyse whole-brain FC patterns without the need for *a priori* information. Here we built FC maps based on SVD analysis. The FC maps corresponded to the spatial singular vector of the spatiotemporal matrix containing the power Doppler signal time profiles of all brain pixels (see Methods). SVD processing decomposed the fUS data into separate space and time variables, without the need for computational optimization or *a priori* information. We searched for the GSMs of the SVD that represented the FC on *N* acquisitions of different animals for each coronal plane. One fUS acquisition could be interpreted as a three-dimensional matrix: a two-dimensional spatial map of vascularization was acquired at multiple time points. We introduced *fUS*(*x*, *z*, *t*) as the spatiotemporal matrix of one fUS acquisition, where *x* and *z* are the spatial variables that describe the lateral distance and depth of one power Doppler image, respectively, and *t* indicates the time at which the power Doppler image was acquired.

The SVD may be thought of as decomposing a matrix into a weighted, ordered sum of the separate matrices. Consequently, the acquisition matrix *fUS*(*x*, *z*, *t*) was written after SVD as:


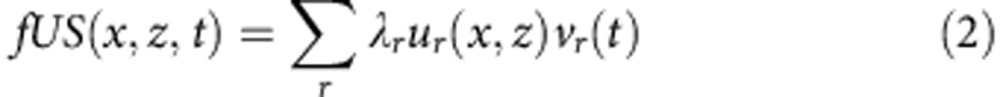


Using SVD, *fUS*(*x*, *z*, *t*) was decomposed into different orthogonal, spatiotemporal representations, each expressed by *u*_r_(*x*, *z*) and *v*_r_(*t*): one spatial image, modulated by a temporal signal, *v*_*r*_(*t*). In other words, all pixels of the image *u*_*r*_(*x*, *z*) behaved with the same fluctuating time signal *v*_*r*_(*t*). The main advantage of this orthogonal representation of the matrix *fUS*(*x*, *z*, *t*) was that each spatiotemporal representation was weighted by *λ*_*r*_; this parameter allows the ranking of these spatiotemporal representations according to their energy weight in the matrix *fUS*(*x*, *z*, *t*). A high value of *λ*_*r*_ means that the associated spatiotemporal representation *u*_*r*_(*x*, *z*) and *v*_*r*_(*t*) are highly representative of the spatiotemporal fluctuations of the matrix, that is, they describe the FC of the brain, whereas a low value of *λ*_*r*_ means that the associated spatiotemporal representation *u*_*r*_(*x*, *z*) and *v*_*r*_(*t*) may represent only noise. Using the SVD on fUS data permits extraction of the main characteristics of the FC by choosing the spatiotemporal representation with the highest associated *λ*_*r*_.

The main remaining problem with SVD is that a clear threshold on the physically relevant values of *λ*_*r*_ is difficult to define with an unbiased approach. For this reason, the method proposed in this article aimed to extract functional brain connectivity patterns directly from the entire population of *N* rats. Using the first SVD of each individual fUS acquisition as a noise filter, we suppressed the noise space associated with the lowest singular values from the spatiotemporal representations of the fUS data of each individual animal. Then, for each coronal plane we integrated the fUS acquisition data of *N* rats and applied a second SVD to this global matrix, representing the combined noise-filtered data of *N* rats to find the GSM. The last processing step consisted of establishing a clear threshold to take advantage of the several *N* fUS acquisitions. The specific steps of the algorithm are detailed in the Methods and are illustrated in Fig. 8.

### Reshaping the fUS acquisitions

For each coronal plane, one fUS acquisition was performed on *N* different rats. One fUS acquisition output is a three-dimensional matrix of power Doppler images of size *N*_X_ (samples on the lateral direction) and *N*_z_ (samples in the depth direction), and acquired at *N*_T_ different times. Figure 8a shows the resulting three-dimensional fUS matrix. We start by changing every three-dimensional acquisition (depth, lateral direction and time) into a two-dimensional matrix by reshaping the space dimensions (depth and lateral direction) on one side (*N*_S_=*N*_X_ × *N*_Z_ samples) and the time dimension on the other side (*N*_T_ samples).

For each fUS acquisition (rat number *i*), we obtain a two-dimensional matrix *A*_*i*_, *i*ε{1,2,…,*N*} for which each column vector represents a Doppler image. The reshaped matrix for the first rat *A*_1_ is displayed in Fig. 8b.

### Centring and normalizing the data

The SVD is based on an energy criterion. Therefore, to take in account all of the scales of spatiotemporal fluctuations, we centre the time profile (mean value of the temporal variation centred to zero) and normalize each time profile of the fUS acquisition. Therefore, for each matrix *A*_*i*_, *i*ε{1,2,…,*N*}, each row vector is centred and normalized. For the sake of clarity, we will still call this matrix *A*_*i*_.

SVD of *A*_*i*_

An SVD for each *A*_*i*_ is performed and this matrix can be written as:


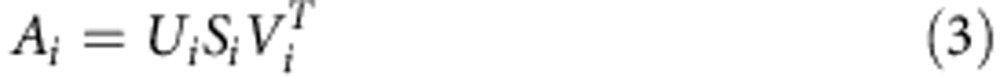


where *T* stands for the matrix transposition, *U*_*i*_ the matrix whose column vectors are the spatial singular vectors of the matrix *A*_*i*_, *V*_*i*_ the matrix whose column vectors are the temporal singular vectors of the matrix *A*_*i*_ and *S*_*i*_ a diagonal matrix of coefficients containing the singular value of the matrix *A*_*i*_. (Note that the number of non-zero diagonal coefficients is the exact rank of the matrix *A*_*i*_). In fact, the *r*^th^ column vector of *U*_*i*_ and *V*_*i*_ can be identified as an image and a time signal, respectively, defining a spatiotemporal representation of the matrix *A*_*i*_ with the *r*^th^ diagonal coefficient of *S*_*i*_, the associated weight.

The results for the first acquisition *U*_1_, *S*_1_ and *V*_1_ of the SVD for the first rat (matrix *A*_1_) are shown in Fig. 9c.

### Noise removal

The number of singular vectors where a non-zero singular value is found is equal to the smallest dimension of the matrix *A*_*i*_. In our case, as the number of spatial samples (*N*_S_~10,000) exceeds the number of temporal samples (*N*_T_=300), we obtain *N*_T_ singular vectors. Most of these vectors contain only noise and are irrelevant for determining the GSM of the brain FC. Therefore, we keep only the *N*_R_ singular vectors that correspond to the highest singular values, and we increase the SNR of these matrices by removing the last singular vectors. The enhanced matrix computed from *A*_*i*_ can be expressed as:


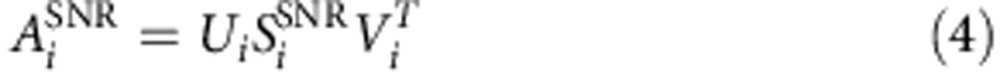


where


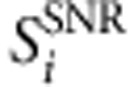
 is a diagonal matrix of the same size as *S*_*i*_ containing only the first *N*_R_ singular value of the matrix *A*_*i*_.

### Extracting spatial modes of FC from fUS acquisitions

If we look at the spatiotemporal characteristics of the FC, we notice that the temporal behaviour of the FC signal is random in different animals; only the spatial characteristics will be conserved. Therefore, to unify the *N* acquisitions from each fUS acquisition, we only keep the highest spatial singular vectors with their weight (singular value), making only the first *N*_R_ vectors of the matrices *U*_*i*_ and 
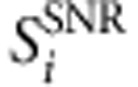
 relevant. From this set of relevant singular vectors, we can compute the matrix 
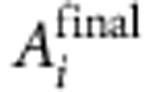
, whose size is *N*_S_ × *N*_R_, for each fUS acquisition. These matrix column vectors are the *N*_R_ first column vectors of *U*_*i*_ multiplied by their singular value contained in the diagonal elements of 
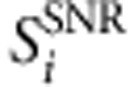
.

### Unifying the information of the N fUS acquisitions

To unify the information over all of the *N* fUS acquisitions, we create a matrix *A*^tot^, which is the concatenation of the 
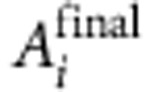
 matrices computed from the fUS acquisitions on the *i*=1 to *N* rodents. *A*^tot^ can be expressed as: 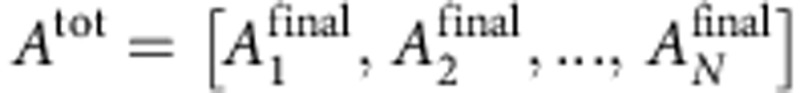. Figure 9d shows this matrix *A*^tot^.

### Computing the GSM of the FC

The GSM of the FC is found using SVD of *A*^tot^. To compute such a process, the line vectors of this matrix must be first centred and normalized as in step 2. For the sake of clarity, we will still call this matrix *A*^tot^. We can decompose the matrix *A*^tot^ as:


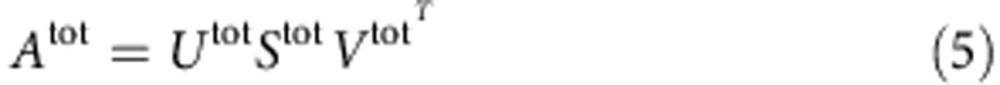


The GSM can be computed within a matrix *M*. These matrix column vectors are the *N*_R_ first column vectors of *U*^tot^ multiplied by their singular value contained in the diagonal elements of *S*^tot^. We only keep the first *N*_R_ column vector of *U*^tot^, because we can consider that there are less relevant global vectors than *N*_R_. The GSM can be computed by reshaping the two-dimensional matrix *M* into a three-dimensional matrix. We performed the opposite process of step 1, except that the third dimension of the reshaped matrix will be the rank of the GSM.

The remaining problem is that *M* contains more column vectors than the number of GSMs representing the FC in the total population of *N* animals. The column vector of *M* can contain specific FC in one animal but only biological noise in other animals, and the last column vectors of *M* can contain only acquisition noise. Therefore, we must find an efficient way to threshold which vector column of *M* is representative for the FC in the total population of *N* animals and can be called the GSM (global meaning relevant for all animals). To measure whether one vector column of *M* is a GSM, we studied its strength in the FC of each animal by projecting it onto each matrix 
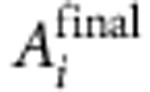
. If this vector is strongly present in each 
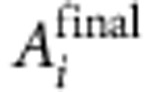
 matrix, it was considered a GSM.

### Projecting the column vector of M on each fUS acquisition

To explain why we project the column vector of *M*, we take the example of the *k*^th^ column vector, which can be expressed as a space vector 
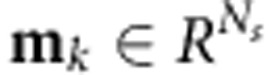
, as this vector has *N*_S_ coefficients. We shall also express the matrix describing the spatial FC of the *i*^th^ rat 
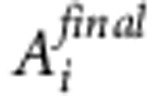
 as vectors: the column *r* of this matrix can be expressed as 
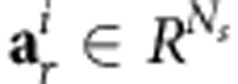
 with *r*ε{1,2,...,*N*_R_} , these *N*_R_ vectors are orthogonal due to the SVD properties.

The main question is how can we measure the strength of the vector **m**_*k*_ among the vectors 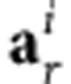, *r*ε{1,2,...,*N*_R_}? We can project the vector **m**_*k*_ using the scalar product and introduce the projection of **m**_*k*_ on the vectors 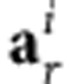, *r*ε{1,2,...,*N*_R_}: 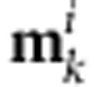. 
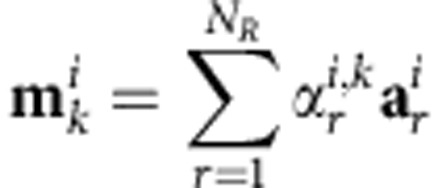
 with 
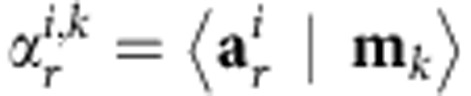
 is the scalar product between the vector 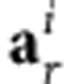 and the vector **m**_*k*_.

Next, we can measure the collinearity of **m**_*k*_ and its projection 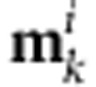 using a normalized scalar product:


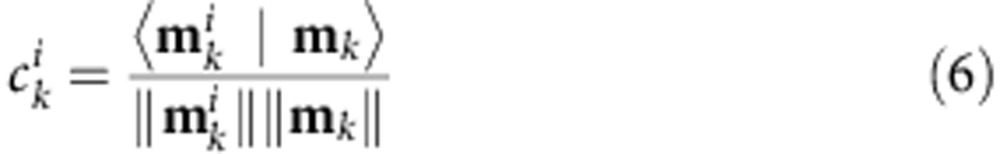


If a space vector **m**_*k*_ is perfectly represented in the FC of the *i*^th^ rat, that is, one 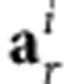 is collinear to **m**_*k*_ so 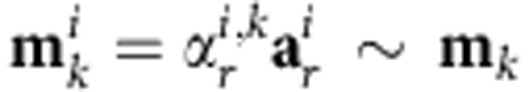, the associated 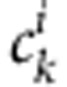 will be equal to 1. If **m**_*k*_ is noise, the value of the associated 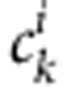 will be lower. We will still have a residual colinearity, because at step seven the **m**_*k*_ are computed from an SVD of the matrix *A*^tot^ containing the 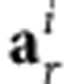, *r*ε{1,2,...,*N*_R_}. As we aim at finding the GSM on *N* rats, we will study the average of 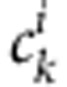 on *N* animals: *c*_*k*_ can be expressed as:


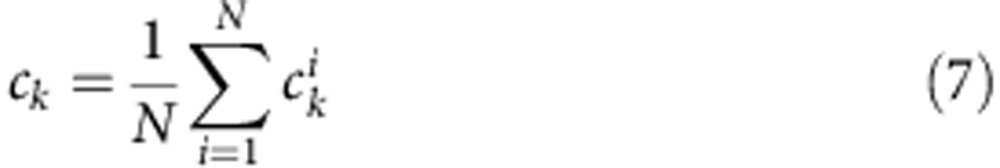


In Fig. 9e, blue dots show the *c*_*k*_ for *k*=1 to *N*_R_ for the coronal level at Bregma −0.6 mm (in this study we chose *N*_R_=150). We notice that the first six coefficients *c*_*k*_ have a high value (>0.6), which means their associated column vector **m**_*k*_ is strongly present in the matrix 
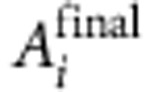
. The *c*_*k*_ for *k* varying from 7 to 35 have a low value that fluctuates. This means that their associated **m**_*k*_ represents some functional characteristic of a few rats over the total *N* rats. As they are not present in all rats, we consider that they are highlighting the biological noise and we do not keep them as relevant singular vectors. Therefore, the last *c*_*k*_ (*k*>35) are due to the acquisition noise.

### Thresholding the c_k_ distribution

To accurately threshold which lowest value of *c*_*k*_ is representative of the global FC over the total *N* rats and is not due to any kind of noise, we can apply the same process (the height first steps) to a set of *N* three-dimensional random matrices (*N*_X_ × *N*_X_ × *N*_Z_) containing only Gaussian random noise. The obtained mean coefficients (
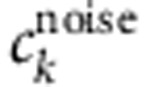
) are displayed by red dots in Fig. 8e. We clearly see that it matches the last *c*_*k*_ (*k*>35) of the fUS experimental acquisitions, because these last are due to the acquisition noise, which can be considered as random. We can select the GSM, which has a 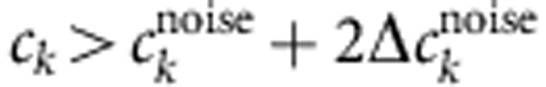, where 
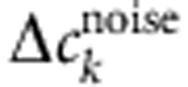
 is the s.d. of 
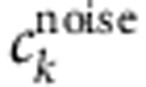
 over *N* rats:


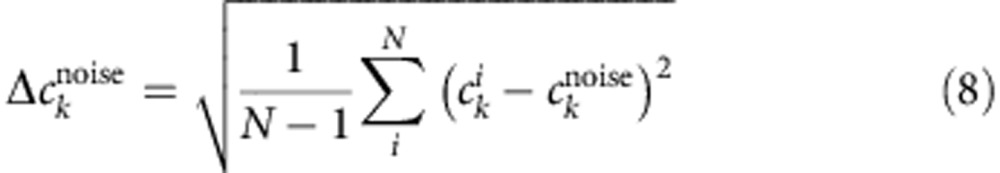


This curve is displayed in Fig. 8e with the red line.

At the end, we select the first six column vectors of *M* to be the GSM of the FC over *N* rats for the coronal slice at Bregma −0.6 mm. These first six GSM are displayed in Fig. 9f. The same process is applied to the other two coronal slices.

## Author contributions

B.O., S.P., Z.L. and M.T. designed the experiments; B.O., S.P. and A.R. performed the experiments; B.O. and S.P. analysed the data; and B.O., S.P., Z.L. and M.T. wrote the paper.

## Additional information

**How to cite this article:** Osmanski, B.-F. *et al*. Functional ultrasound imaging of intrinsic connectivity in the living rat brain with high spatiotemporal resolution. *Nat. Commun.* 5:5023 doi: 10.1038/ncomms6023 (2014).

## References

[b1] Biswal B., Yetkin F. Z., Haughton V. M. & Hyde J. S. Functional connectivity in the motor cortex of resting human brain using echo-planar MRI. Magn. Reson. Med. 34, 537–541 (1995). 8524021 10.1002/mrm.1910340409

[b2] Hutchison R. M. . Dynamic functional connectivity: promise, issues, and interpretations. Neuroimage 80, 360–378 (2013). 23707587 10.1016/j.neuroimage.2013.05.079 PMC3807588

[b3] Raichle M. E. The restless brain. Brain Connect. 1, 3–12 (2011). 22432951 10.1089/brain.2011.0019 PMC3621343

[b4] Buckner R. L., Ndrews-Hanna J. R. & Schacter D. L. The brain’s default network: anatomy, function, and relevance to disease. Ann. N. Y. Acad. Sci. 1124, 1–38 (2008). 18400922 10.1196/annals.1440.011

[b5] Corbetta M. & Shulman G. L. Control of goal-directed and stimulus-driven attention in the brain. Nat. Rev. Neurosci. 3, 201–215 (2002). 11994752 10.1038/nrn755

[b6] Fox M. D., Corbetta M., Snyder A. Z., Vincent J. L. & Raichle M. E. Spontaneous neuronal activity distinguishes human dorsal and ventral attention systems. Proc. Natl Acad. Sci. USA 103, 10046–10051 (2006). 16788060 10.1073/pnas.0604187103 PMC1480402

[b7] Seeley W. W. . Dissociable intrinsic connectivity networks for salience processing and executive control. J. Neurosci. 27, 2349–2356 (2007). 17329432 10.1523/JNEUROSCI.5587-06.2007 PMC2680293

[b8] Larson-Prior L. J. . Cortical network functional connectivity in the descent to sleep. Proc. Natl Acad. Sci. USA 106, 4489–4494 (2009). 19255447 10.1073/pnas.0900924106 PMC2657465

[b9] Hutchison R. M., Womelsdorf T., Gati J. S., Everling S. & Menon R. S. Resting-state networks show dynamic functional connectivity in awake humans and anesthetized macaques. Hum. Brain Mapp. 34, 2154–2177 (2013). 22438275 10.1002/hbm.22058 PMC6870538

[b10] Fox M. D. & Raichle M. E. Spontaneous fluctuations in brain activity observed with functional magnetic resonance imaging. Nat. Rev. Neurosci. 8, 700–711 (2007). 17704812 10.1038/nrn2201

[b11] Greicius M. Resting-state functional connectivity in neuropsychiatric disorders. Curr. Opin. Neurol. 21, 424–430 (2008). 18607202 10.1097/WCO.0b013e328306f2c5

[b12] Menon V. Large-scale brain networks and psychopathology: a unifying triple network model. Trends Cogn. Sci. 15, 483–506 (2011). 21908230 10.1016/j.tics.2011.08.003

[b13] Lu H. . Rat brains also have a default mode network. Proc. Natl Acad. Sci. USA 109, 3979–3984 (2012). 22355129 10.1073/pnas.1200506109 PMC3309754

[b14] Birn R. M., Diamond J. B., Smith M. A. & Bandettini P. A. Separating respiratory-variation-related fluctuations from neuronal-activity-related fluctuations in fMRI. Neuroimage 31, 1536–1548 (2006). 16632379 10.1016/j.neuroimage.2006.02.048

[b15] Shmueli K. . Low-frequency fluctuations in the cardiac rate as a source of variance in the resting-state fMRI BOLD signal. Neuroimage 38, 306–320 (2007). 17869543 10.1016/j.neuroimage.2007.07.037 PMC2128785

[b16] Tanter M. & Fink M. Ultrafast imaging in biomedical ultrasound 1. IEEE Trans. Ultrason. Ferroelectr. Freq. Control. 61, 102–119 (2014). 24402899 10.1109/TUFFC.2014.6689779

[b17] Mace E. . Functional ultrasound imaging of the brain. Nat. Methods 8, 662–664 (2011). 21725300 10.1038/nmeth.1641

[b18] Lagraoui M. . Controlled cortical impact and craniotomy induce strikingly similar profiles of inflammatory gene expression, but with distinct kinetics. Front. Neurol. 3, 155 (2012). 23118733 10.3389/fneur.2012.00155 PMC3484408

[b19] Forcelli P. A., Kalikhman D. & Gale K. Delayed effect of craniotomy on experimental seizures in rats. PLoS ONE 8, e81401 (2013). 24324691 10.1371/journal.pone.0081401 PMC3852486

[b20] Cole J. T. . Craniotomy: true sham for traumatic brain injury, or a sham of a sham? J. Neurotrauma 28, 359–369 (2011). 21190398 10.1089/neu.2010.1427 PMC3057208

[b21] Kaas J. H. inEpilepsy and the Corpus Callosum 2. Advances in Behavioral Biology eds Reeves A. G., Roberts D. W. 15–27Plenum Press (1995).

[b22] Paxinos G. & Watson C. The Rat Brain in Strereotaxic Coordinates Academic Press (2006).

[b23] Hutchison R. M., Mirsattari S. M., Jones C. K., Gati J. S. & Leung L. S. Functional networks in the anesthetized rat brain revealed by independent component analysis of resting-state FMRI. J. Neurophysiol. 103, 3398–3406 (2010). 20410359 10.1152/jn.00141.2010

[b24] Liang Z., King J. & Zhang N. Uncovering intrinsic connectional architecture of functional networks in awake rat brain. J. Neurosci. 31, 3776–3783 (2011). 21389232 10.1523/JNEUROSCI.4557-10.2011 PMC3073070

[b25] Yeo B. T. . The organization of the human cerebral cortex estimated by intrinsic functional connectivity. J. Neurophysiol. 106, 1125–1165 (2011). 21653723 10.1152/jn.00338.2011 PMC3174820

[b26] Hutchison R. M. . Resting-state networks in the macaque at 7 T. Neuroimage 56, 1546–1555 (2011). 21356313 10.1016/j.neuroimage.2011.02.063

[b27] Liang Z., King J. & Zhang N. Intrinsic organization of the anesthetized brain. J. Neurosci. 32, 10183–10191 (2012). 22836253 10.1523/JNEUROSCI.1020-12.2012 PMC3422560

[b28] Schwarz A. J. . Anti-correlated cortical networks of intrinsic connectivity in the rat brain. Brain Connect. 3, 503–511 (2013). 23919836 10.1089/brain.2013.0168 PMC3796325

[b29] De Luca M., Beckmann C. F., De Stefano N., Matthews P. M. & Smith S. M. fMRI resting state networks define distinct modes of long-distance interactions in the human brain. Neuroimage 29, 1359–1367 (2006). 16260155 10.1016/j.neuroimage.2005.08.035

[b30] Murphy K., Birn R. M. & Bandettini P. A. Resting-state fMRI confounds and cleanup. Neuroimage 80, 349–359 (2013). 23571418 10.1016/j.neuroimage.2013.04.001 PMC3720818

[b31] Osmanski B. F. . Functional ultrasound imaging reveals different odor-evoked patterns of vascular activity in the main olfactory bulb and the anterior piriform cortex. Neuroimage 95, 176–184 (2014). 24675645 10.1016/j.neuroimage.2014.03.054

[b32] Fox M. D. . The human brain is intrinsically organized into dynamic, anticorrelated functional networks. Proc. Natl Acad. Sci. USA 102, 9673–9678 (2005). 15976020 10.1073/pnas.0504136102 PMC1157105

[b33] Liang Z., King J. & Zhang N. Anticorrelated resting-state functional connectivity in awake rat brain. Neuroimage 59, 1190–1199 (2012). 21864689 10.1016/j.neuroimage.2011.08.009 PMC3230741

[b34] Murphy K., Birn R. M., Handwerker D. A., Jones T. B. & Bandettini P. A. The impact of global signal regression on resting state correlations: are anti-correlated networks introduced? Neuroimage 44, 893–905 (2009). 18976716 10.1016/j.neuroimage.2008.09.036 PMC2750906

[b35] Fox M. D., Zhang D., Snyder A. Z. & Raichle M. E. The global signal and observed anticorrelated resting state brain networks. J. Neurophysiol. 101, 3270–3283 (2009). 19339462 10.1152/jn.90777.2008 PMC2694109

[b36] Carbonell F., Bellec P. & Shmuel A. Quantification of the impact of a confounding variable on functional connectivity confirms anti-correlated networks in the resting-state. Neuroimage 86, 343–353 (2014). 24128734 10.1016/j.neuroimage.2013.10.013

[b37] Biswal B. B., Eldreth D. A., Motes M. A. & Rypma B. Task-dependent individual differences in prefrontal connectivity. Cereb. Cortex 20, 2188–2197 (2010). 20064942 10.1093/cercor/bhp284 PMC2923215

[b38] Arieli A., Sterkin A., Grinvald A. & Aertsen A. Dynamics of ongoing activity: explanation of the large variability in evoked cortical responses. Science 273, 1868–1871 (1996). 8791593 10.1126/science.273.5283.1868

[b39] Goldman R. I., Stern J. M., Engel J. Jr & Cohen M. S. Simultaneous EEG and fMRI of the alpha rhythm. Neuroreport 13, 2487–2492 (2002). 12499854 10.1097/01.wnr.0000047685.08940.d0 PMC3351136

[b40] Laufs H. . EEG-correlated fMRI of human alpha activity. Neuroimage 19, 1463–1476 (2003). 12948703 10.1016/s1053-8119(03)00286-6

[b41] Cabral J. . Structural connectivity in schizophrenia and its impact on the dynamics of spontaneous functional networks. Chaos 23, 046111 (2013). 24387590 10.1063/1.4851117

[b42] Shanahan M. The brain’s connective core and its role in animal cognition. Philos. Trans. R. Soc. Lond. B Biol. Sci. 367, 2704–2714 (2012). 22927569 10.1098/rstb.2012.0128 PMC3427545

[b43] Deco G., Jirsa V. K. & McIntosh A. R. Emerging concepts for the dynamical organization of resting-state activity in the brain. Nat. Rev. Neurosci. 12, 43–56 (2011). 21170073 10.1038/nrn2961

[b44] Fox M. D., Buckner R. L., White M. P., Greicius M. D. & Pascual-Leone A. Efficacy of transcranial magnetic stimulation targets for depression is related to intrinsic functional connectivity with the subgenual cingulate. Biol. Psychiatry 72, 595–603 (2012). 22658708 10.1016/j.biopsych.2012.04.028 PMC4120275

[b45] Chai X. J., Castanon A. N., Ongur D. & Whitfield-Gabrieli S. Anticorrelations in resting state networks without global signal regression. Neuroimage 59, 1420–1428 (2012). 21889994 10.1016/j.neuroimage.2011.08.048 PMC3230748

[b46] Chang C. & Glover G. H. Effects of model-based physiological noise correction on default mode network anti-correlations and correlations. Neuroimage 47, 1448–1459 (2009). 19446646 10.1016/j.neuroimage.2009.05.012 PMC2995588

[b47] Logothetis N. K. What we can do and what we cannot do with fMRI. Nature 453, 869–878 (2008). 18548064 10.1038/nature06976

[b48] Liu T. T. Neurovascular factors in resting-state functional MRI. Neuroimage 80, 339–348 (2013). 23644003 10.1016/j.neuroimage.2013.04.071 PMC3746765

[b49] Hoge R. D. . Linear coupling between cerebral blood flow and oxygen consumption in activated human cortex. Proc. Natl Acad. Sci. USA 96, 9403–9408 (1999). 10430955 10.1073/pnas.96.16.9403 PMC17795

[b50] Davis T. L., Kwong K. K., Weisskoff R. M. & Rosen B. R. Calibrated functional MRI: mapping the dynamics of oxidative metabolism. Proc. Natl Acad. Sci. USA 95, 1834–1839 (1998). 9465103 10.1073/pnas.95.4.1834 PMC19199

[b51] Biswal B. B., Van K. J. & Hyde J. S. Simultaneous assessment of flow and BOLD signals in resting-state functional connectivity maps. NMR Biomed. 10, 165–170 (1997). 9430343 10.1002/(sici)1099-1492(199706/08)10:4/5<165::aid-nbm454>3.0.co;2-7

[b52] Wu C. W. . Mapping functional connectivity based on synchronized CMRO2 fluctuations during the resting state. Neuroimage 45, 694–701 (2009). 19280693 10.1016/j.neuroimage.2008.12.066 PMC2775537

[b53] Fukunaga M. . Metabolic origin of BOLD signal fluctuations in the absence of stimuli. J. Cereb. Blood Flow Metab. 28, 1377–1387 (2008). 18382468 10.1038/jcbfm.2008.25

[b54] Golanov E. V., Yamamoto S. & Reis D. J. Spontaneous waves of cerebral blood flow associated with a pattern of electrocortical activity. Am. J. Physiol 266, R204–R214 (1994). 8304543 10.1152/ajpregu.1994.266.1.R204

[b55] Vern B. A., Schuette W. H., Leheta B., Juel V. C. & Radulovacki M. Low-frequency oscillations of cortical oxidative metabolism in waking and sleep. J. Cereb. Blood Flow Metab. 8, 215–226 (1988). 2830291 10.1038/jcbfm.1988.52

[b56] Aguirre G. K., Detre J. A., Zarahn E. & Alsop D. C. Experimental design and the relative sensitivity of BOLD and perfusion fMRI. Neuroimage 15, 488–500 (2002). 11848692 10.1006/nimg.2001.0990

[b57] Tjandra T. . Quantitative assessment of the reproducibility of functional activation measured with BOLD and MR perfusion imaging: implications for clinical trial design. Neuroimage 27, 393–401 (2005). 15921936 10.1016/j.neuroimage.2005.04.021

[b58] Liu T. T. & Brown G. G. Measurement of cerebral perfusion with arterial spin labeling: Part 1. Methods. J. Int. Neuropsychol. Soc. 13, 517–525 (2007). 17445301 10.1017/S1355617707070646

[b59] Miller K. L. . Nonlinear temporal dynamics of the cerebral blood flow response. Hum. Brain Mapp. 13, 1–12 (2001). 11284042 10.1002/hbm.1020 PMC6871988

[b60] Wang J. . Arterial spin labeling perfusion fMRI with very low task frequency. Magn. Reson. Med. 49, 796–802 (2003). 12704760 10.1002/mrm.10437

[b61] Boas D. A., Dale A. M. & Franceschini M. A. Diffuse optical imaging of brain activation: approaches to optimizing image sensitivity, resolution, and accuracy. Neuroimage 23(Suppl 1), S275–S288 (2004). 15501097 10.1016/j.neuroimage.2004.07.011

[b62] Nasiriavanaki M. . High-resolution photoacoustic tomography of resting-state functional connectivity in the mouse brain. Proc. Natl Acad. Sci. USA 111, 21–26 (2014). 24367107 10.1073/pnas.1311868111 PMC3890828

[b63] Demene C. . Ultrafast Doppler reveals the mapping of cerebral vascular resistivity in neonates. J. Cereb. Blood Flow Metab. 34, 1009–1017 (2014). 24667916 10.1038/jcbfm.2014.49 PMC4050246

[b64] Upadhyay J. . Default-mode-like network activation in awake rodents. PLoS ONE 6, e27839 (2011). 22125628 10.1371/journal.pone.0027839 PMC3220684

[b65] Raichle M. E. Cognitive neuroscience. Bold insights. Nature 412, 128–130 (2001). 11449247 10.1038/35084300

[b66] Yang G., Pan F., Parkhurst C. N., Grutzendler J. & Gan W. B. Thinned-skull cranial window technique for long-term imaging of the cortex in live mice. Nat. Protoc. 5, 201–208 (2010). 20134419 10.1038/nprot.2009.222 PMC4690457

[b67] Pawela C. P. . A protocol for use of medetomidine anesthesia in rats for extended studies using task-induced BOLD contrast and resting-state functional connectivity. Neuroimage 46, 1137–1147 (2009). 19285560 10.1016/j.neuroimage.2009.03.004 PMC2693293

[b68] Zhao F., Zhao T., Zhou L., Wu Q. & Hu X. BOLD study of stimulation-induced neural activity and resting-state connectivity in medetomidine-sedated rat. Neuroimage 39, 248–260 (2008). 17904868 10.1016/j.neuroimage.2007.07.063 PMC2137163

[b69] Li Y. . Direct labeling and visualization of blood vessels with lipophilic carbocyanine dye DiI. Nat. Protoc. 3, 1703–1708 (2008). 18846097 10.1038/nprot.2008.172 PMC2811090

[b70] Bercoff J. . Ultrafast compound Doppler imaging: providing full blood flow characterization. IEEE Trans. Ultrason. Ferroelectr. Freq. Control 58, 134–147 (2011). 21244981 10.1109/TUFFC.2011.1780

